# Metformin promotes osteogenic differentiation of human periodontal ligament stem cells via KLF2-mediated activation of miR-181a-5p under lipopolysaccharide stimulation

**DOI:** 10.1007/s13577-025-01262-3

**Published:** 2025-07-15

**Authors:** Xu Zhang, Wen Hu, Hua Hua

**Affiliations:** Department of Emergency and General Dentistry, Changsha Stomatological Hospital, No. 389 Youyi Road, Tianxin District, Changsha, 41000 Hunan Province People’s Republic of China

**Keywords:** Periodontitis, Tissue engineering, Stem cells, MicroRNA, Regeneration

## Abstract

Periodontal ligament stem cells (PDLSCs) constitute a promising source for successful periodontal regeneration. This study aims to explore roles of metformin, krüppel-like factor 2 (KLF2), and miR-181a-5p in mediating osteogenic differentiation of human PDLSCs (hPDLSCs) following lipopolysaccharide (LPS) stimulation. The osteogenic differentiation potential of hPDLSCs isolated from human premolar root samples were examined by alkaline phosphatase (ALP) staining, ALP activity assay, Alizarin red S staining, and Western blotting of osteogenic markers. Metformin pretreatment at dose of 100 μM significantly resulted in increased ALP activity, elevated protein expressions of osteogenic markers, and more generated mineralized matrix in hPDLSCs with LPS stimulation. KLF2 and miR-181a-5p were found to be increased by metformin pretreatment at dose of 100 μM in hPDLSCs with stimulation but not in hPDLSCs without LPS stimulation. The interaction between the KLF2 and the promoter of miR-181a-5p was noted by the dual-luciferase reporter assay. KLF2 knockdown or miR-181a-5p inhibition notably abrogated the improvements of osteogenic differentiation by metformin pretreatment in LPS-stimulated hPDLSCs. The findings of the study indicate metformin protects hPDLSCs against impaired osteogenic differentiation of hPDLSCs after LPS stimulation by KLF2-mediated activation of miR-181a-5p under inflammation conditions.

## Introduction

Periodontal bone regeneration is a major challenge in the treatment of periodontitis [1]. Stem cell-based tissue engineering has been emerged as the key component of periodontal tissue engineering technology to repair periodontal supporting tissues [2]. Periodontal ligament stem cells (PDLSCs) exhibit strong self-renewal abilities and multidirectional differentiation potential, are concerned as the most ideal cell source among dental stem cells for tissue engineering to repair the degeneration of periodontal ligament [3]. The osteogenic differentiation potential of PDLSCs is of great significance for successful periodontal regeneration, creating a critical need to maintain or enhance this differentiation of PDLSCs by gene-addition/editing therapy or combing with the usage of biomaterials [4,5].

Metformin is the most widely prescribed drug for treating type 2 diabetes, which also has shown to outstandingly prevent age-associated inflammation [6]. Recently, a metformin-resin has been found to considerably enhance osteogenic and cementogenic differentiation of human PDLSCs (hPDLSCs), producing more than twofold mineral synthesis than control without metformin [7]. Injectable and self-setting calcium phosphate cement scaffold delivering hPDLSCs and metformin demonstrated excellent efficacy for dental and craniofacial applications [8]. Krüppel-like factor 2 (KLF2), a transcription factor, has been extensively studied as an important indicator of the stemness of human mesenchymal stem cells (hMSCs) and KLF2 + stemness-maintained hMSCs show more efficiently osteogenic differentiation potential [9]. KLF2 overexpression could promote neuronal differentiation and osteogenic differentiation of dental pulp-derived stem cells by inducing mitophagy and autophagy [10,11]. Earlier work has demonstrated metformin increased KLF2 expression and inhibited inflammatory response, thus exerting antiatherogenic effects [12]. A previous study reported KLF2 induced exosomal miR-181a-5p to attenuate pulmonary vascular remodeling and prevent pulmonary arterial hypertension [13]. miR-181a-5p was found to be downregulated in gingival crevicular fluid samples and saliva samples obtained from patients with periodontitis, and this downregulation was associated with the severity of periodontitis [14]. Bone marrow MSCs secrete exosomes harboring miR-181a-5p exhibited anti-inflammatory activity [15]. miR-181a-5p was upregulated in the developing palatal shelves in areas of bone formation, indicating its role in enhancing early osteogenic differentiation [16]. Accordingly, we proposed an interesting hypothesis that PDLSCs benefit with improved osteogenic differentiation in the inflammation conditions from metformin treatment and this improvement may be achieved by KLF2 mediated transcription regulation of miR-181a-5p. To prove this hypothesis, we mimicked an inflammatory condition in hPDLSCs by lipopolysaccharide (LPS) stimulation to investigate the roles of metformin, KLF2, and miR-181a-5p in osteogenic differentiation of hPDLSCs.

## Materials and methods

### Isolation and phenotype analysis of hPDLSCs

Four healthy premolars extracted from 4 donors (aged 14–24) during orthodontic treatments were collected for isolation of hPDLSCs. The use of premolars received informed consent signed by the donor or their legal guardians. The premolar extraction was approved by the Ethics Committee of our hospital (No. 2023–96). The periodontal ligament tissues were scraped off from the middle root of the extracted premolars using a sterile scalpel and placed into the centrifuge tube in the super-clean worktable. Following treatment with phosphate buffered saline (PBS, Corning Inc., New York, USA) containing 3 mg/mL collagenase I (Sigma Aldrich, St. Louis, MO, USA) for 40 min and shaking at a 10 min interval, the tube was added with α-MEM (Gibco, Grand Isalnd, NY, USA) containing 10% fetal bovine serum (FBS) (Gibco) to terminate collagenase I treatment. After centrifugation at 700 × g for 10 min for removal of the supernatant, the tube was added with α-MEM containing 20% FBS. The mixture in the tube was inoculated into a culture flask which was placed in the incubator (37 °C, 5%CO_2_, saturated humidity), with the medium replaced every 3 days. The hPDLSCs at passages 3 were characterized by fluorescence-activated cell sorting (FACS) analysis using a CytoFLEX (Beckman Coulter, Brea, California, USA) with phycoerythrin-conjugated monoclonal antibodies against CD44, CD105, and HLA-DR (Cyagen, Guangzhou, China).

### Multidifferentiation assays of hPDLSCs

For osteogenic potential detection of hPDLSCs, the hPDLSCs at passages 3 were treated with pancreatin to make single cell suspension. The suspension was inoculated into a 6-well plate (2 × 10^5^/well) and cultured with osteogenic induction medium consisting of α-MEM with 10% FBS (Gibco), 50 μg/ml ascorbic acid (Solarbio, Beijing, China), 10 mmol/l β-glycerophosphate sodium (Solarbio), and 100 nmol/l dexamethasone (Solarbio). The osteogenic induction medium was refreshed every 3 days. After 21 days, the cells were stained with 2% Alizarin red S (Sigma Aldrich). For adipogenic potential detection of hPDLSCs, the 6-well plate inoculated with 2 × 10^5^ hPDLSCs each well was cultured with the adipogenic induction medium consisting of α-MEM medium containing 10% FBS (Gibco), 1 µM dexamethasone (Solarbio), 0.5 mM 3-isobutyl-1-methylxanthine (Sigma Aldrich), and 10 μg/mL insulin (Solarbio). The adipogenic induction medium was refreshed every 3 days. After 28 days, the cells were stained with oil red O (Sigma Aldrich).

### Cell treatments

For cell pretreatment, hPDLSCs were cultured in complete culture medium containing 10-, 100-, 1000-, and 2000-μM metformin (Solarbio) for 24 h. KLF2 was knocked down in hPDLSCs using small interfering RNA (siRNA) against KLF2 (5'-AUUUUGAAAAACAAAACUCGUGAGUUUUGUUUUUCAAAAUGG-3'), with negative control siRNA (si-NC, 5'‑CCUGGCGCCUUCGGUCUUUUU‑3') used as control. KLF2 overexpression in hPDLSCs was achieved by transfecting with the recombinant pcDNA3.1(-)/KLF2 cDNA vector, with vector-null (oe-NC) used as control. All plasmids were purchased from GenePharma (Shanghai, China). The lipofectamine 3000 reagents (Invitrogen, Carlsbad, CA, USA) were utilized as the transfection reagent as per the procedures provided by the manufacturer. To induce inflammation conditions, hPDLSCs were stimulated with 1 μg/mL lipopolysaccharide (LPS) (Sigma Aldrich) for 24 h after metformin treatment and plasmid transfection [17].

### Alkaline phosphatase (ALP) staining and ALP activity assay

The ALP staining was performed using a BCIP/NBT Alkaline Phosphatase Color Development Kit (Beyotime, Shanghai, China). Briefly, the hPDLSCs were seeded in 12-well plates (10^5^ cells per well) and cultured in the osteogenic induction medium concurrent with different treatments. The osteogenic induction medium was refreshed every three days. After 14 day osteogenic induction, the hPDLSCs were PBS-raised twice and paraformaldehyde-fixed, followed by addition of BCIP/NBT solution. After staining for 6 h, the hPDLSCs stained by ALP were captured with the aid of an Olympus BX-53 microscope (Tokyo, Japan). To determine the ALP activity, protein extraction was performed in hPDLSCs undergoing 14-day osteogenic induction and analyzed using an Alkaline Phosphatase Assay Kit (Beyotime).

### Alizarin red S staining and quantification

Following dual PBS washes and treatment of paraformaldehyde, the hPDLSCs undergoing 14-day osteogenic induction concurrent with different treatments were stained with 2% Alizarin red S (Sigma Aldrich). After staining for 6 h, the mineralized nodules formed were captured with the aid of an Olympus BX-53 light microscope and then desorbed by addition of 10% cetylpyridinium chloride (Sigma‐Aldrich, USA) to quantify the staining (optical density at 570 nm).

### CCK-8 assays

The hPDLSCs were seeded in a 96-well plate (5,000 cells for each well) and cultured in α-MEM medium containing 10% FBS in the presence of different concentrations of metformin (0, 10, 100, 1000, and 2000 μM). Following an incubation at three time points (24, 48 and 72 h), each well in the plate was added with 10 uL of CCK-8 reagent (Beyotime) and incubated for 4 h. Using a microplate reader for absorbance measurement at 450 nm.

### Western blotting

The hPDLSCs reacted in the RIPA lysis buffer to obtain total protein, followed by sodium dodecyl sulfate–polyacrylamide gel electrophoresis (10%) separation, transfer onto polyvinylidene fluoride membrane, and blocking buffer treatment (5% skim milk in TBST). The membranes were incubated with anti-KLF2 (MA5-26,825, Invitrogen), anti-RUNX2 (#2556, Cell Signaling Technology, Beverly, MA, USA), anti-OPN (MA5-17,180, Invitrogen), and anti-β-actin (#4967, Cell Signaling Technology), followed by rinse with TBST and incubation with rabbit anti-human IgG (MA5-42,729, Invitrogen). The signal of immunoblots was visualized using an ECL chromogenic kit (Beyotime) and β-actin normalized the density of each immunoblot.

### Immunofluorescence

After rinsing with PBS, the hPDLSCs were fixed with 4% paraformaldehyde for 15 min and permeabilized with 0.5% Triton X-100 for 20 min, followed by incubation with 5% bovine serum albumin (BSA) for 30 min to block non-specific antigen. Subsequently, the hPDLSCs were incubated with anti-KLF2 antibody (MA5-26,825, Invitrogen), washed with PBS, and incubated with fluorescein isothiocyanate-conjugated goat anti‑human IgG (Cat. No. SA00003-12; ProteinTech Group, Inc., Wuhan, China). After DAPI staining for cel nuclei for 2 h, the cells were rinsed again with PBS and mounted. Images were captured under a fluorescence microscope (Carl Zeiss, Oberkochen, Germany).

### Quantitative real-time PCR (qRT-PCR)

Extraction of total RNA from the hPDLSCs and the following reverse transcription into cDNA were completed using Trizol reagent (Invitrogen) and PrimeScript RT reagent (Takara, Tokyo, Japan), respectively. The mRNA expression of KLF2 was determined using SYBR Premix Ex Taq (Takara). The miRNA purification kit (iGeneBio, Guangzhou, China) and the miRNA qRT-PCR detection kit (iGeneBio) were utilized for miRNA extraction, miRNA reverse transcription and qRT-PCR detection. The qRT-PCR was performed on a Bio-Rad CFX96 PCR system (Bio-Rad, Hercules, CA, USA). The resulting KLF2 mRNA and miR-181a-5p expressions were normalized GAPDH or U6 levels, and calculated using the 2^−∆∆Ct^ method. Used primer sequences are: 5'-CACCAAGAGTTCGCATCTGA-3'(forward) and 5'-CATGTGCCGTTTCATGTG-3'(reverse) for KLF2; 5'-AACAUUCAACGCUGUCGGUGAGU-3'(forward) and 5'-UCACCGACAGCGUUGAAUGUUUU-3'(reverse) for miR-181a-5p; 5'-GGAGCGAGATCCCTCCAAAAT-3'(forward) and 5'-GGCTGTTGTCATACTTCTCATGG-3'(reverse) for GAPDH; 5'-AACGCTTCACGAATTTGCGT-3'(forward) and 5'-CTCGCTTCGGCAGCACA-3'(reverse) for U6.

### Luciferase reporter assay

The miR-181a-5p promoter four fragments (wt, mut#1–3) containing the three binding sites (wt: wild type on site 1, 2, and 3; mut#1: wild type on sit 1, mutant on sit 2 and 3; mut#2: wild type on site 2, mutant on site 1 and 3; mut#3: wild type on site 3, mutant on site 1 and 2) of KLF2 were established based on the JASPAR database (https://jaspar.elixir.no/). The four fragments were amplified and inserted into the pGL3-Basic reporter vectors (Promega, Madison, WI, USA) between the Kpn I and Xho I site to establish recombinant luciferase reporter plasmids. Then, these luciferase reporter vectors were co-transfected with si-KLF2 into HEK-293 T cells. The Dual-Glo Luciferase system (Promega) was employed to examine the luciferase activity and the pRL-TK activity normalized the results.

### Statistical analysis

Results of mean with standard deviation (s.d.) were yielded from six biological and technical replicates. Unpaired t test, One-way analysis of variance (ANOVA) plus Tukey’s post-hoc test, and two-way ANOVA plus Bonferroni post-hoc test were performed with the aid of GraphPad Prism version 8.0 (GraphPad Software, La Jolla, CA, USA) for Windows. A value of *p* < 0.05 was regarded statistically significant.

## Results

### Identification of hPDLSCs

**Fig. 1:**
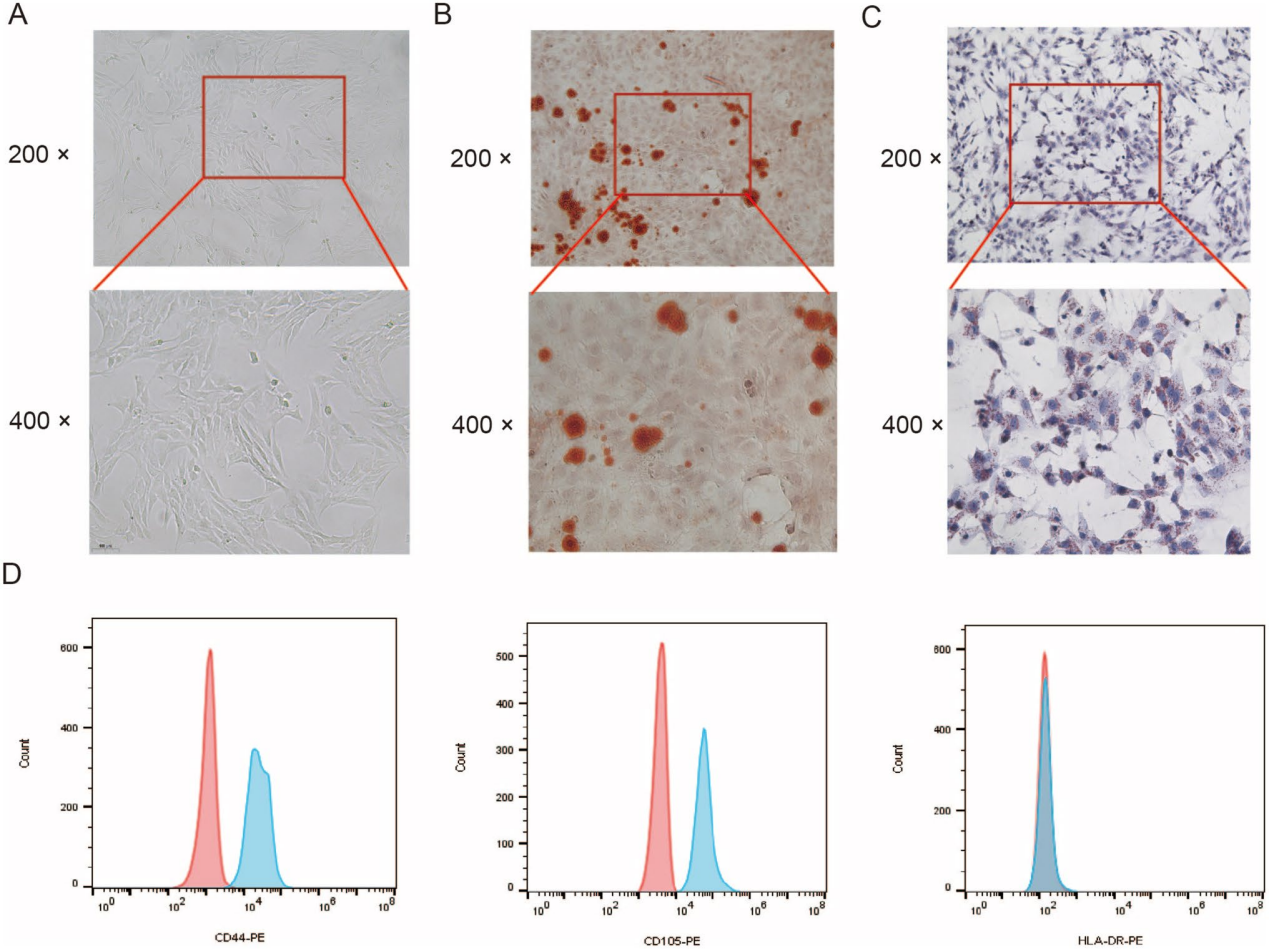
Identification of hPDLSCs. **A**, Representative images (200 ×; 400 ×) of hPDLSC subculture. **B**, Representative images (200 ×; 400 ×) of osteogenic differentiation of hPDLSCs evaluated by Alizarin red S staining. **C**, Representative images (200 ×; 400 ×) of adipogenic differentiation of hPDLSCs evaluated by oil red O staining. **D**, The phenotype analysis of hPDLSCs using flow cytometry

The primary cells merged from the edge of the periodontal tissue block after 5–7 days of cell culture, most of which presented a long spindle shape. After subculture, the cells rapidly grew and arranged in bundles, with full cell body and clear nucleus (Fig. 1A). In addition, the multidifferentiation potential of isolated cells was demonstrated as evidenced by the presence of Alizarin red-stained nodules (Fig. 1B) and oil red O-stained lipid droplets (Fig. 1C). The results of flow cytometry analysis indicated the isolated cells were positive for CD44 and CD105 as two known MSC markers and negative for HLA-DR as a hematopoietic-specific cell marker (Fig. 1D). Therefore, the hPDLSCs were successfully obtained.

### Metformin promotes osteogenic differentiation in LPS-stimulated hPDLSCs

**Fig. 2:**
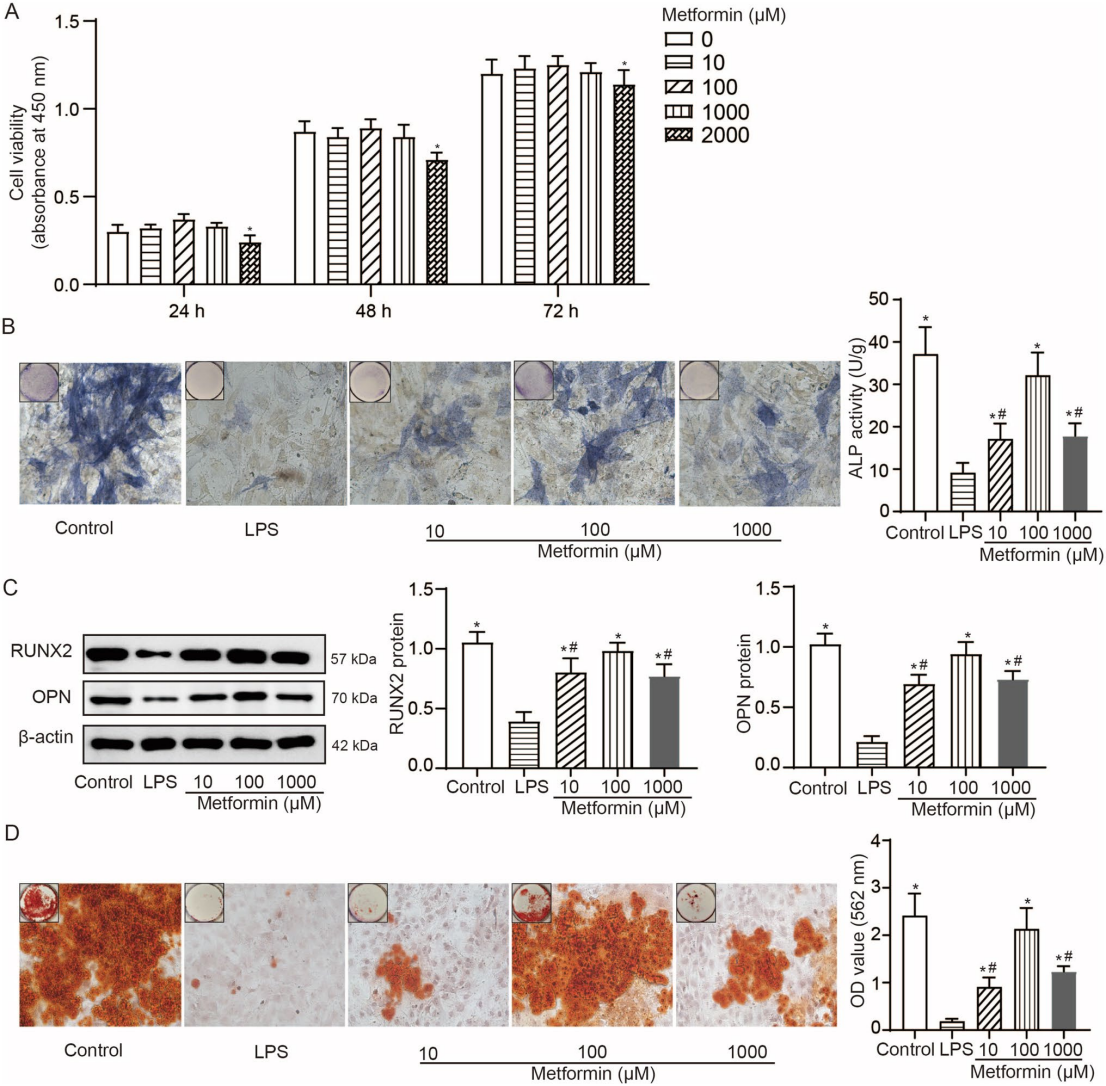
Metformin promotes osteogenic differentiation in LPS-stimulated hPDLSCs. **A**, CCK-8 assays to detect the viability of hPDLSCs after 10-, 100-, 1000- and 2000-μM metformin treatment for 24, 48, and 72 h; results of mean with s.d. were yielded from six biological and technical replicates and analyzed by two-way ANOVA plus Bonferroni post-hoc test; *P < 0.05 compared to other concentrations of metformin. **B**, Representative images (400 ×) of ALP-positive cells and the ALP activity in LPS-stimulated hPDLSCs with 10-, 100-, and 1000-μM metformin pretreatment after 14 day osteogenic induction. **C**, Western blotting detections of RUNX2 and OPN in LPS-stimulated hPDLSCs with 10-, 100-, and 1000-μM metformin pretreatment after 7 day osteogenic induction. **D**, Representative images (400 ×) of Alizarin red-stained nodules and the quantitative analysis (OD value at 562 nm) of mineralized matrix in LPS-stimulated hPDLSCs with 10-, 100-, and 1000-μM metformin pretreatment after 21 day osteogenic induction. For panel **B-D**, results of mean with s.d. were yielded from six biological and technical replicates and analyzed by one-way ANOVA plus Tukey’s post-hoc test. *P < 0.05 compared to LPS-stimulated hPDLSCs without metformin pretreatment and #*P* < 0.05 compared to LPS-stimulated hPDLSCs with 100 μM metformin pretreatment

Firstly, we used different concentrations of metformin (0, 10, 100, 1000, and 2000 μM) to treat hPDLSCs and performed CCK-8 assays. Results of CCK-8 assays (Fig. 2A) showed a reduced cell viability in hPDLSCs after treatment with 2000 μM metformin. To mimick the inflammation condition, we treated hPDLSCs with 1 μg/mL LPS. To study the protection of metformin on osteogenic differentiation of hPDLSCs against LPS stimulation, we pre-treated hPDLSCs with metformin (10, 100, and 1000 μM) prior to LPS stimulation. The results of ALP staining (Fig. 2B), Western blotting (Fig. 2C), and Alizarin red S staining (Fig. 2D) showed increased ALP activity, elevated protein expressions of RUNX2, OPN, and more generated mineralized matrix in LPS-stimulated hPDLSCs with metformin pretreatment compared to no pretreatment. These effects induced by 100-μM metformin pretreatment were more significantly than 10- and 1000-μM metformin pretreatment in LPS-stimulated hPDLSCs.

### Metformin increases KLF2 expression in hPDLSCs under inflammation condition

**Fig. 3:**
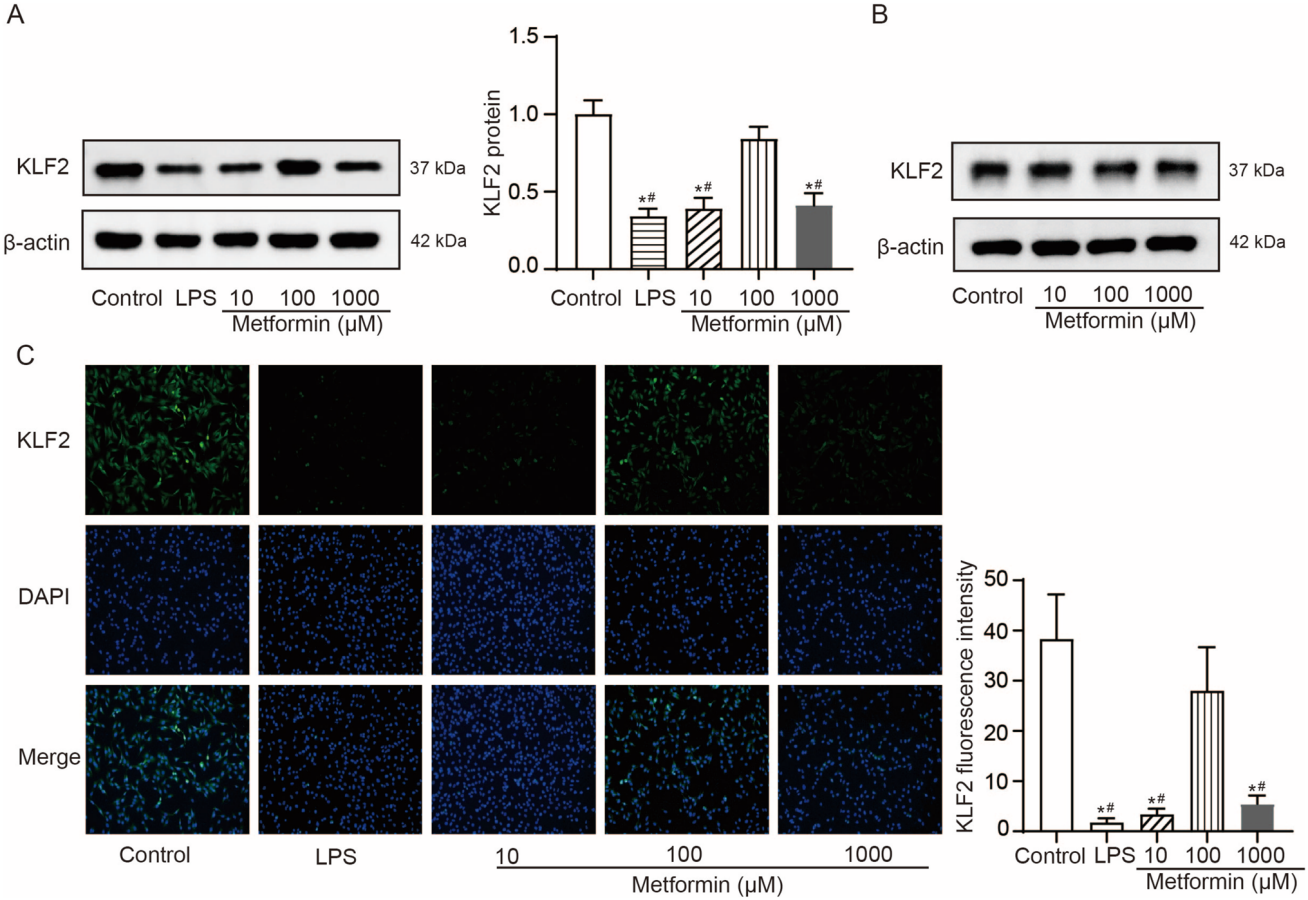
Metformin increases KLF2 expression in hPDLSCs under inflammation condition. **A**, Western blotting detections of KLF2 protein in control hPDLSCs, LPS-stimulated hPDLSCs with or without 10-, 100-, and 1000-μM metformin pretreatment. **B**, Western blotting detections of KLF2 protein in control hPDLSCs with 10-, 100-, and 1000-μM metformin treatment. C, Representative images (200 ×) showing immunofluorescence staining of the intracellular localization of KLF2 in control hPDLSCs, LPS-stimulated hPDLSCs with or without 10-, 100-, and 1000-μM metformin pretreatment. Results of mean with s.d. were yielded from six biological and technical replicates and analyzed by one-way ANOVA plus Tukey’s post-hoc test; *P < 0.05 compared to control hPDLSCs and #*P* < 0.05 compared to LPS-stimulated hPDLSCs with 100 μM metformin pretreatment

We next investigated whether metformin increasing osteogenic differentiation of hPDLSCs was associated with KLF expression. KLF2 was found to be declined in hPDLSCs treated with 1 μg/mL LPS for 24 h compared to control, and this decline was significantly improved by 100 μM metformin pretreatment but not by 10 μM or 1000 μM metformin pretreatment (Fig. 3A). In hPDLSCs without LPS stimulation, KLF2 did not significantly differ after 10-, 100-, and 1000-μM metformin pretreatment for 24 h, indicating KLF2 may be involved in the positive effects of metformin on hPDLSCs in the inflammation conditions (Fig. 3B). As shown by immunofluorescence analysis, a loss of KLF2 nuclear translocation was noted in hPDLSCs treated with 1 μg/mL LPS for 24 h compared to control, and this loss was significantly prevented by 100 μM metformin pretreatment but not by 10 μM or 1000 μM metformin pretreatment (Fig. 3C).

### The effects of metformin on LPS-stimulated hPDLSCs are KLF2-dependent

**Fig. 4:**
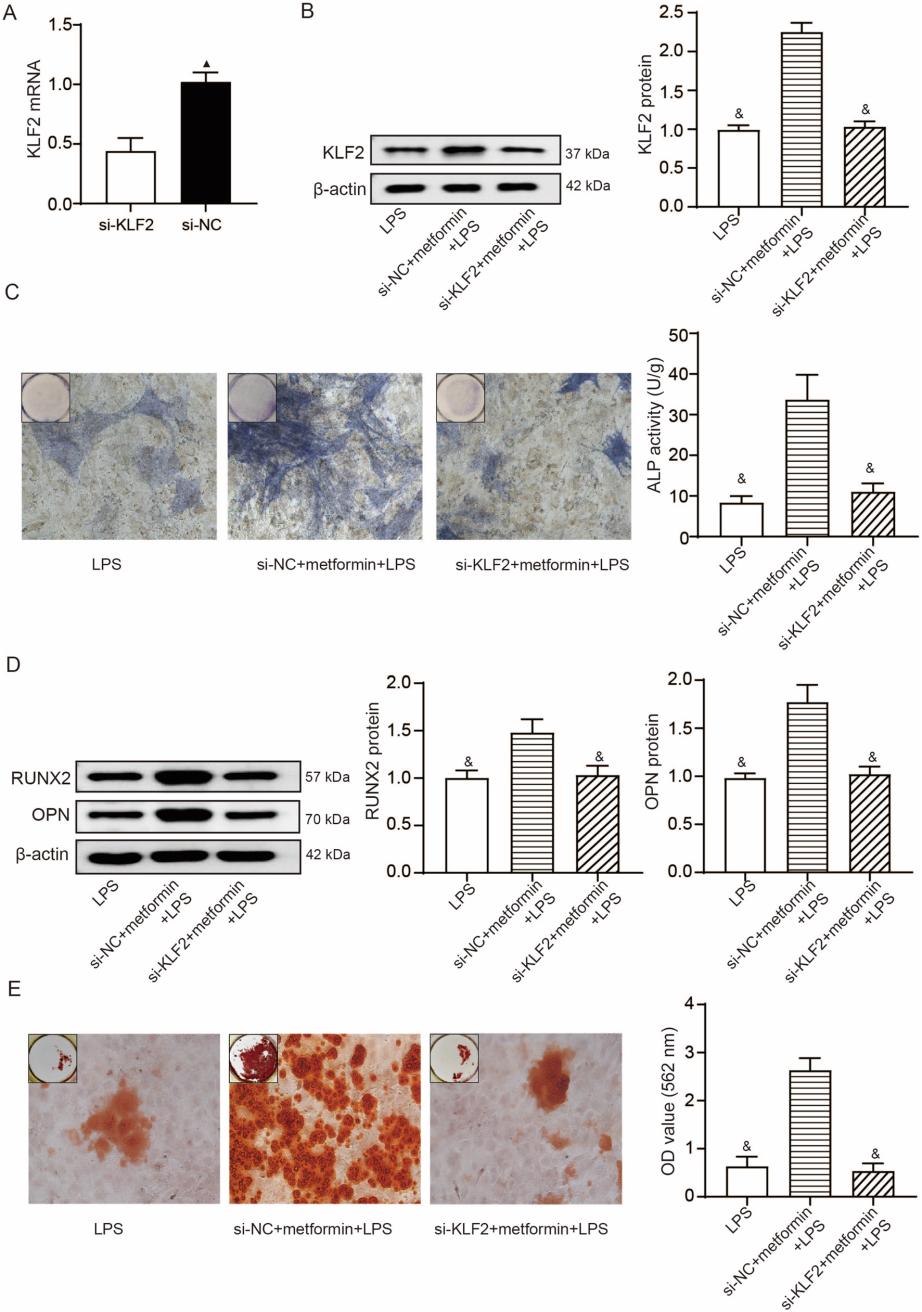
The effects of metformin on LPS-stimulated hPDLSCs are KLF2-dependent. **A**, The efficiency of KLF2 knockdown by si-KLF2, determined by qRT-PCR; results of mean with s.d. were yielded from six biological and technical replicates and analyzed by unpaired t test; ▲ P < 0.05 compared to si-KLF2. **B**, Western blotting detections of KLF2 protein in LPS-stimulated hPDLSCs with or without si-KLF2 pre-transfection and metformin pretreatment. **C**, Representative images (400 ×) of ALP-positive cells and the ALP activity in LPS-stimulated hPDLSCs with or without si-KLF2 pre-transfection and metformin pretreatment after 14 day osteogenic induction. **D**, Western blotting detections of RUNX2 and OPN in LPS-stimulated hPDLSCs with or without si-KLF2 pre-transfection and metformin pretreatment. E, Representative images (400 ×) of Alizarin red-stained nodules and the quantitative analysis (OD value at 562 nm) of mineralized matrix in LPS-stimulated hPDLSCs with or without si-KLF2 pre-transfection and metformin pretreatment. &*P* < 0.05 compared to si-NC + metformin + LPS. For panel **B-E**, results of mean with s.d. were yielded from six biological and technical replicates and analyzed by one-way ANOVA plus Tukey’s post-hoc test

We genetically knocked down KLF2 in hPDLSCs, and the results of qRT-PCR demonstrated KLF2 was significantly inhibited by si-KLF2 treatment in hPDLSCs compared to si-NC (Fig. 4A). The hPDLSCs with KLF2 knockdown continued to be treated by 100-μM metformin for 24 h and 1 μg/mL LPS for another 24 h. Results of Western blotting showed the si-KLF2 + metformin + LPS group exhibited a reduced expression of KLF2 protein compared to the si-NC + metformin + LPS group (Fig. 4B). The following results obtained from ALP staining, Western blotting, and Alizarin red S staining revealed reductions in ALP activity, expressions of RUNX2 and OPN proteins, and mineralized matrix formation in the si-KLF2 + metformin + LPS group compared to the si-NC + metformin + LPS group (Fig. 4C-E).

### KLF2-mediated activation of miR-181a-5p is involved in metformin effects on LPS-stimulated hPDLSCs

**Fig. 5:**
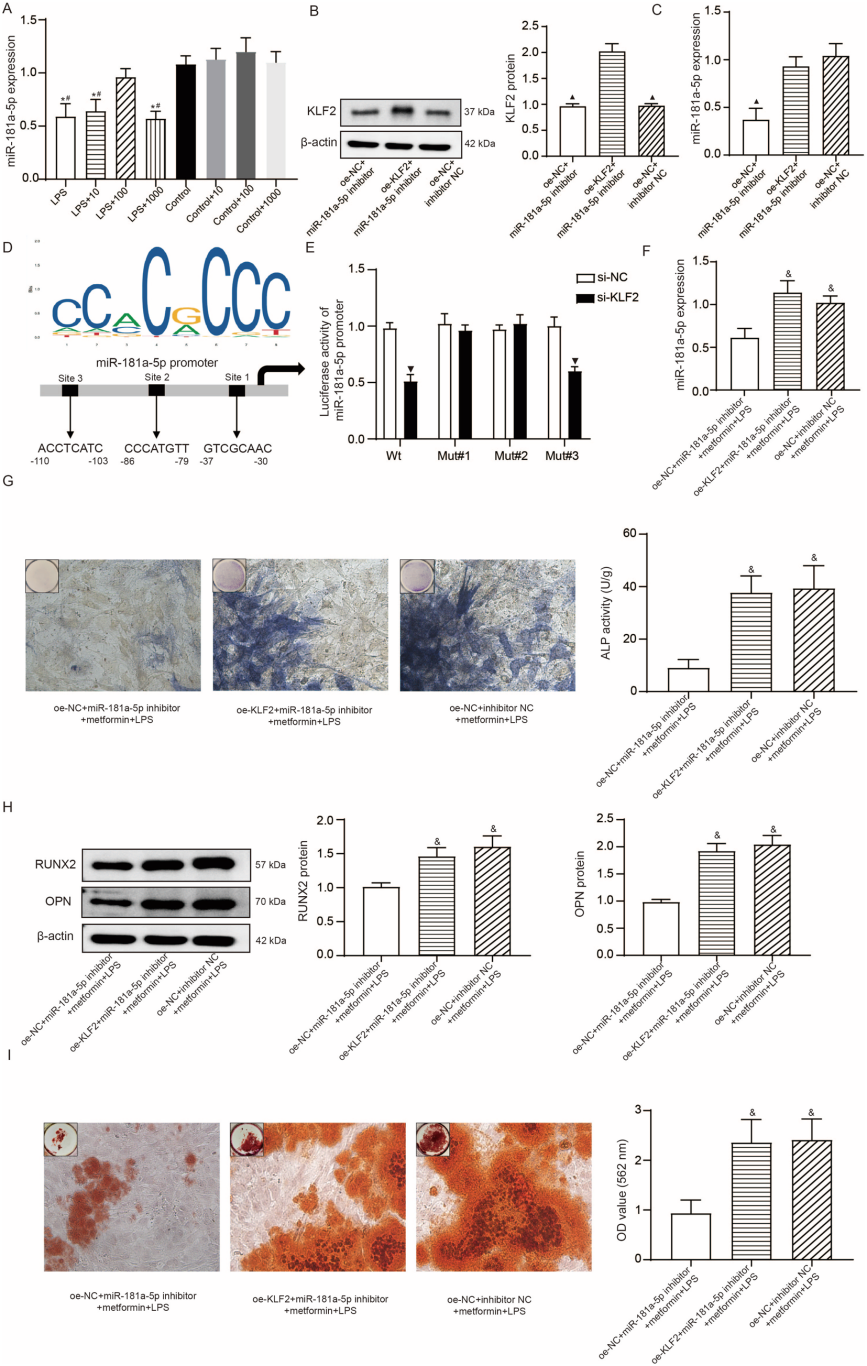
KLF2-mediated activation of miR-181a-5p is involved in metformin effects on LPS-stimulated hPDLSCs. **A**, The qRT-PCR detections of miR-181a-5p in control hPDLSCs with or without 10-, 100-, and 1000-μM metformin treatment, in LPS-stimulated hPDLSCs with or without 10-, 100-, and 1000-μM metformin pretreatment; *P < 0.05 compared to control hPDLSCs and #*P* < 0.05 compared to LPS-stimulated hPDLSCs with 100 μM metformin pretreatment. **B**, Western blotting detections of KLF2 protein in control hPDLSCs transfected with oe-KLF2 and miR-181a-5p inhibitor. **C**, The qRT-PCR detections of miR-181a-5p in control hPDLSCs transfected with oe-KLF2 and miR-181a-5p inhibitor; ▲ *P* < 0.05 compared to oe-KLF2 + miR-181a-5p inhibitor. **D**, Three putative binding positions of KLF2 in the miR-181a-5p promoter based on the JASPAR database analysis and named as sites 1–3. **E**, Dual-luciferase reporter assay was performed to confirm the target area of KLF2 in the promoter of miR-181a-5p; results of mean with s.d. were yielded from six biological and technical replicates and analyzed by unpaired t test; ▼ *P* < 0.05 compared to si-NC. **F**, The qRT-PCR detections of miR-181a-5p in LPS-stimulated hPDLSCs with or without oe-KLF2, miR-181a-5p inhibitor pre-transfection, and metformin pretreatment. **G**, Representative images (400 ×) of ALP-positive cells and the ALP activity in LPS-stimulated hPDLSCs with or without oe-KLF2, miR-181a-5p inhibitor pre-transfection, and metformin pretreatment after 14-day osteogenic induction. **H**, Western blotting detections of RUNX2 and OPN in LPS-stimulated hPDLSCs with or without oe-KLF2, miR-181a-5p inhibitor pre-transfection, and metformin pretreatment after 7 day osteogenic induction. **I**, Representative images (400 ×) of Alizarin red-stained nodules and the quantitative analysis (OD value at 562 nm) of mineralized matrix in LPS-stimulated hPDLSCs with or without oe-KLF2, miR-181a-5p inhibitor pre-transfection, and metformin pretreatment after 21 day osteogenic induction. & *P* < 0.05 compared to oe-NC + miR-181a-5p inhibitor + metformin + LPS. For panel **A-C** and **F-I**, results of mean with s.d. were yielded from six biological and technical replicates and analyzed by one-way ANOVA plus Tukey’s post-hoc test

To ascertain the mechanism behind KLF2 regulation of osteogenic differentiation of hPDLSCs in the inflammation conditions, we focused on changes of miR-181a-5p in response to KLF2 knockdown and metformin treatment. Results of qRT-PCR showed a decreased expression of miR-181a-5p in hPDLSCs treated with 1 μg/mL LPS compared to control. Metformin pretreatment notably prevented LPS-caused downregulation of miR-181a-5p at dose of 100 μM but not at 10 μM or 1000 μM (Fig. 5A). In hPDLSCs without LPS stimulation, metformin pretreatment at 10- and 100-μM did not significantly affect the expression of miR-181a-5p. Subsequently, we treated hPDLSCs with oe-KLF2 and/or miR-181a-5p inhibitor. The results of Western blotting and qRT-PCR demonstrated KLF2 overexpression in hPDLSCs transfected with oe-KLF2 and miR-181a-5p inhibition in hPDLSCs transfected with miR-181a-5p inhibitor (Fig. 5B, C). It was also found that transfection of oe-KLF2 significantly led to an increased miR-181a-5p expression in hPDLSCs in the presence of miR-181a-5p inhibitor. The JASPAR database analysis (Fig. 5D) revealed the binding site of the transcription factor KLF2, and there are three possible binding sites between KLF2 and the promoter sequence of miR-181a-5p. Treatment with si-KLF2 could inhibit the luciferase activity of cells driven by wt or mut#3, but not mut#1 and mut#2, indicating KLF2 bound to the promoter of miR-181a-5p at site 1 and 2 (Fig. 5E). These transfected cells continued to be treated by 100-μM metformin for 24 h and 1 μg/mL LPS for another 24 h. Compared to oe-KLF2 + miR-181a-5p inhibitor + metformin + LPS group and si-NC + inhibitor NC + metformin + LPS group, oe-NC + miR-181a-5p inhibitor + metformin + LPS group showed reductions in miR-181a-5p expression (Fig. 5F), ALP activity (Fig. 5G), expressions of RUNX2 and OPN proteins (Fig. 5H), and mineralized matrix formation (Fig. 5I).

## Discussion

Developing methods to maintain or enhance the resistance of PDLSCs to disordered immune microenvironment is important for PDLSC-based bone tissue engineering. In this study, we pretreated hPDLSCs with metformin and found metformin at a concentration of 100 μM protects hPDLSCs against impaired osteogenic differentiation of hPDLSCs after LPS stimulation by KLF2-mediated activation of miR-181a-5p under inflammation conditions.

Maintenance or improvement of functional characteristics, such as osteogenic differentiation, of stem cells by metformin has been extensively studied [18,19]. In the dental field focusing on periodontal tissue engineering technology, metformin has been demonstrated with the ability to facilitate osteogenic differentiation of stem cells from human exfoliated deciduous teeth and PDLSCs [20,21]. The positive effects of metformin on osteogenic differentiation of hPDLSCs against high glucose- or H_2_O_2_-induced damage have been consistently reported in earlier work [22,23]. Although inflamed hPDLSCs exhibit similarities in terms of differentiation and immunomodulatory properties comparable to healthy hPDLSCs, overwhelmed inflammation in the context of periodontitis still impairs stem cell functionalities of resident hPDLSCs, particularly their osteogenic properties [24,25]. In cell viability assays, we found low concentrations of metformin (less than 1000 µM) did not significantly affect the viability of hPDLSCs, but the cell viability was inhibited when the concentration reached 2000 µM, creating a critical need of the optimal concentration of metformin to its application in tissue engineering. Although no significant difference was found in the viability of hPDLSCs after 10-, 100-, and 1000-μM metformin treatment, treatment with 10 and 1000 μM metformin showed reduced expressions of osteogenesis-related genes and osteogenic differentiation compared to 100 μM metformin under inflammation conditions. The one reason may be different concentrations of metformin exhibiting different effects on hPDLSCs in different microenvironments, normal conditions and inflammatory conditions. In inflammatory microenvironment, as the concentration increases to 1000 μM, the drug may have other unexpected effects, such as affecting cell energy metabolism, leading to a reduction in its ability of osteogenic differentiation. These inconsistent results indicate that the effect of metformin might vary depending on the concentration and microenvironment. Our results of only 100-μM metformin promoting KLF2 expression in inflamed hPDLSCs while 1000-μM metformin failing to change KLF2 expression suggest different concentrations of metformin influence different mechanisms. As reported by Bian et al., they found a low concentration of metformin downregulated Akt, p44/42 MAPK, JNK, p38 MAPK phosphorylation, while a high concentration only ERK and Akt phosphorylation during osteoclast differentiation [26]. In our study, the KLF2-denepdent mechanism in 100-μM metformin treatment may partially explain less potent potential of 1000-μM metformin treatment in inflammatory microenvironment.

Patients experiencing either acute or chronic inflammation exhibit an approximately 30% to 50% reduction in KLF2 levels [27]. Earlier work has demonstrated LPS stimulation caused a decrease in KLF2 expression by inducing DNMT1-mediated hypermethylation in its promoter region [28]. In addition, LPS stimulation reduced the expression of myocyte enhancer factor 2 (MEF2) which was necessary for the KLF2 expression [29]. Recent literature showed metformin eliminated LPS and tumor necrosis factor-⍺ (TNF-⍺) caused downregulation of KLF2 but did not affect the expression of KLF2 in endothelial cells without treatment of inflammatory stimuli [30]. This content suggested metformin only affected the expression of KLF2 under inflammation conditions, which aligned with our data. AMP-activated protein kinase (AMPK)-dependent pathways play a key role in cellular and molecular mechanisms of metformin, which explain metformin leading to activation of AMPK targets including KLF2 [31,32]. In atherosclerosis, metformin could increase the expressions of KLF2 and nuclear erythroid-related factor 2 (Nrf2) [33]. In two recent reports [34,35], KLF2 mediated activation of Nrf2 provides protection to endothelial cells or vascular smooth muscle cells. KLF2 could promote the nuclear import of Nrf2 and prime the antioxidant activity [36]. Jia et al. [23] demonstrated metformin enhanced osteogenic differentiation and protects against oxidative stress-induced damage in PDLSCs by the activation of Nrf2 signaling pathway, which added evidence to prove the regulation of metformin on KLF2 expression to the promote osteoblast differentiation of hPDLSCs. Just as shown by our data, we did not determine significant changes of KLF2 expressions in response to 10- and 100-μM metformin pretreatment in hPDLSCs without LPS stimulation. The anti-inflammatory, antioxidant role of KLF2 have been reported in previous studies [37,38]. Although not in PDLSCs, Maity et al. proved the positive effects of KLF2 on osteoblast differentiation from dental pulp-derived stem cells [11]. The roles of KLF2 in maintaining or enhancing stemness and self-renewal of human bone marrow MSCs were demonstrated in Gong et al.’s study [39] and Wu et al.’s study [40]. KLF2 has been found to regulate runt-related transcription factor 2 (RUNX2) which is a predominant transcription factor involving in osteoblast differentiation and bone mineralization [41].

The mechanism behind KLF2’s involvement in metformin effects was also a focus of our study. KLF2 can transcriptionally regulate miRNAs, such as miR-181a-5p and miR-324-5p, in the pathogenesis of human disease [13]. Our study observed KLF2-mediatede activation of miR-181a-5p and overexpression of miR-181a-5p could promote the osteogenic differentiation of hPDLSCs. However, there are some discrepancies in current reports concerning the role of miR-181a-5p in the osteogenic differentiation of mesenchymal stem cells. In line with our results, Schoen et al. [16] demonstrated an increased expression of miR-181a-5p during early osteogenic differentiation in the developing palatal shelves in areas of bone formation. Similarly, Long et al. [42] found that the transcription level of miR-181a-5p was evidently enhanced during the osteogenic differentiation of mouse MC3T3-E1 preosteoblast cells, and its inhibition aggravated osteoporosis in old mice. Including but not limited to the study of Xu et al. [43], the researchers demonstrated the nano-hydroxyapatite scaffold loaded with miR-181a-5p-modified MSC could effectively facilitate the repair of bone defects via enhancing osteogenic differentiation. Inconsistent with our results, Hong et al. have reported a decreased expression of miR-181a-5p triggered by metformin treatment, suggesting downregulation of miR-181a-5p may be associated with improved osteogenic ability of aging human bone marrow MSCs. They studied doses of metformin at 500, 750, and 1000 μM while our studied doses of metformin at 10, 100, and 1000 μM. Further investigations using metformin at dose of 500, 750, and 1000 μM to treat human hPDLSCs to validate the role of miR-181a-5p in the osteogenic differentiation of hPDLSCs. Liu et al. [44] also supported the role of miR-181a-5p in inhibiting the osteogenic differentiation of MC3T3-E1 cells, which was opposite to the results yielded by Guo et al. ^45^ who demonstrated miR-181a-5p promoted the osteogenic differentiation of MC3T3-E1 cells. Considering much debate focusing on the effects of miR-181a-5p on the osteogenic differentiation of stem cells, high-throughput sequencing technologies may be further needed to examine the changes of miR-181a-5p in MC3T3-E1 cells or MSCs during osteogenic induction.

In conclusion, the findings demonstrate metformin pretreatment could provide considerable protection against impaired osteogenic ability to hPDLSCs after LPS stimulation. These beneficial effects of metformin were partly mediated by KLF2-mediatede activation of miR-181a-5p. Considering easier accessibility and availability of inflamed hPDLSCs than healthy hPDLSCs, the study suggests KLF2 may be a promising target to promote the therapeutic efficiency of inflamed hPDLSCs in cellular therapy for successful periodontal regeneration. Combining metformin and cell sheet technology of hPDLSC pretreated by recombinant human KLF2 can be a new direction to repair damaged periodontal complex. The lack of in vivo studies, such as using immunocompromised mice models, limits the clinical utility of KLF2 during PDLSC-based tissue regeneration, which should be centered in further studies.

## Data Availability

No/Not applicable (this manuscript does not report data generation or analysis).

## References

[1] Fischer NG, de Souza Araujo IJ, Daghrery A, et al. Guidance on biomaterials for periodontal tissue regeneration: fabrication methods, materials and biological considerations. Dent Mater. 2025;41:283–305. 39794220 10.1016/j.dental.2024.12.019 PMC13022934

[2] Wang X, Chen J, Tian W. Strategies of cell and cell-free therapies for periodontal regeneration: the state of the art. Stem Cell Res Ther. 2022;13:536. 36575471 10.1186/s13287-022-03225-z PMC9795760

[3] Wen S, Zheng X, Yin W, et al. Dental stem cell dynamics in periodontal ligament regeneration: from mechanism to application. Stem Cell Res Ther. 2024;15:389. 39482701 10.1186/s13287-024-04003-9 PMC11526537

[4] Trubiani O, Pizzicannella J, Caputi S, et al. Periodontal Ligament stem cells: current knowledge and future perspectives. Stem Cells Dev. 2019;28:995–1003. 31017047 10.1089/scd.2019.0025

[5] Liang Q, Du L, Zhang R, Kang W, Ge S. Stromal cell-derived factor-1/Exendin-4 cotherapy facilitates the proliferation, migration and osteogenic differentiation of human periodontal ligament stem cells in vitro and promotes periodontal bone regeneration in vivo. Cell Prolif. 2021;54:e12997. 33511708 10.1111/cpr.12997 PMC7941242

[6] Bailey CJ. Metformin: Therapeutic profile in the treatment of type 2 diabetes. Diabetes Obes Metab. 2024;26(Suppl 3):3–19. 38784991 10.1111/dom.15663

[7] Yu K, Zhu M, Dai Z, et al. Dental resin for periodontal and tooth root regeneration via metformin to enhance osteogenic and cementogenic differentiation of human periodontal ligament stem cells. J Dent. 2024;153:105507. 39643264 10.1016/j.jdent.2024.105507

[8] Sun Y, Zhao Z, Qiao Q, et al. Injectable periodontal ligament stem cell-metformin-calcium phosphate scaffold for bone regeneration and vascularization in rats. Dent Mater. 2023;39:872–85. 37574338 10.1016/j.dental.2023.07.008

[9] Zhou Y, Liu C, He J, et al. KLF2(+) stemness maintains human mesenchymal stem cells in bone regeneration. Stem Cells. 2020;38:395–409. 31721356 10.1002/stem.3120

[10] Prateeksha P, Naidu P, Das M, Barthels D, Das H. KLF2 regulates neural differentiation of dental pulp-derived stem cells by modulating autophagy and mitophagy. Stem Cell Rev Rep. 2023;19:2886–900. 37642902 10.1007/s12015-023-10607-0

[11] Maity J, Barthels D, Sarkar J, et al. Ferutinin induces osteoblast differentiation of DPSCs via induction of KLF2 and autophagy/mitophagy. Cell Death Dis. 2022;13:452. 35552354 10.1038/s41419-022-04903-9 PMC9098908

[12] Wu H, Feng K, Zhang C, et al. Metformin attenuates atherosclerosis and plaque vulnerability by upregulating KLF2-mediated autophagy in apoE(-/-) mice. Biochem Biophys Res Commun. 2021;557:334–41. 33915432 10.1016/j.bbrc.2021.04.029

[13] Sindi HA, Russomanno G, Satta S, et al. Therapeutic potential of KLF2-induced exosomal microRNAs in pulmonary hypertension. Nat Commun. 2020;11:1185. 32132543 10.1038/s41467-020-14966-x PMC7055281

[14] Li Q, Zhu JJ. Expression levels of miR-181 family members in oral biofluids as biomarkers for periodontitis severity. Tohoku J Exp Med. 2024;264:121–30. 38960640 10.1620/tjem.2024.J058

[15] Li H, Du R, Xiang A, Liu Y, Guan M, He H. Bone marrow mesenchymal stem cell-derived exosomal miR-181a-5p promotes M2 macrophage polarization to alleviate acute pancreatitis through ZEB2-mediated RACK1 ubiquitination. FASEB J. 2024;38:e70042. 39614664 10.1096/fj.202400803RR

[16] Schoen C, Bloemen M, Carels CEL, et al. A potential osteogenic role for microRNA-181a-5p during palatogenesis. Eur J Orthod. 2023;45:575–83. 37454242 10.1093/ejo/cjad037 PMC10756689

[17] Ma R, Wang M, Shi P, et al. Effect of lipoxin A4 on the osteogenic differentiation of periodontal ligament stem cells under lipopolysaccharide-induced inflammatory conditions. Eur J Oral Sci. 2023;131:e12932. 37074297 10.1111/eos.12932

[18] Fan S, Zhang C, Sun X, et al. Metformin enhances osteogenic differentiation of BMSC by modulating macrophage M2 polarization. Sci Rep. 2024;14:20267. 39217251 10.1038/s41598-024-71318-1 PMC11365931

[19] Manochantr S, Meesuk L, Chadee N, Suwanprateeb J, Tantrawatpan C, Kheolamai P. Improvement of osteogenic differentiation potential of placenta-derived mesenchymal stem cells by metformin via AMPK pathway activation. Stem Cell Res Ther. 2024;15:417. 39533406 10.1186/s13287-024-04014-6 PMC11559138

[20] Zhao X, Pathak JL, Huang W, et al. Metformin enhances osteogenic differentiation of stem cells from human exfoliated deciduous teeth through AMPK pathway. J Tissue Eng Regen Med. 2020;14:1869–79. 33049108 10.1002/term.3142

[21] Zhang R, Liang Q, Kang W, Ge S. Metformin facilitates the proliferation, migration, and osteogenic differentiation of periodontal ligament stem cells in vitro. Cell Biol Int. 2020;44:70–9. 31293042 10.1002/cbin.11202

[22] Zhang YL, Liu F, Li ZB, et al. Metformin combats high glucose-induced damage to the osteogenic differentiation of human periodontal ligament stem cells via inhibition of the NPR3-mediated MAPK pathway. Stem Cell Res Ther. 2022;13:305. 35841070 10.1186/s13287-022-02992-z PMC9284897

[23] Jia L, Xiong Y, Zhang W, Ma X, Xu X. Metformin promotes osteogenic differentiation and protects against oxidative stress-induced damage in periodontal ligament stem cells via activation of the Akt/Nrf2 signaling pathway. Exp Cell Res. 2020;386:111717. 31715142 10.1016/j.yexcr.2019.111717

[24] Zhang Z, Deng M, Hao M, Tang J. Periodontal ligament stem cells in the periodontitis niche: inseparable interactions and mechanisms. J Leukoc Biol. 2021;110:565–76. 34043832 10.1002/JLB.4MR0421-750R

[25] Li C, Wang X, Tan J, Wang T, Wang Q. The immunomodulatory properties of periodontal ligament stem cells isolated from inflamed periodontal granulation. Cells Tissues Organs. 2014;199:256–65. 25471814 10.1159/000367986

[26] Bian F, Zhang Y, Xie Y, et al. Effects of different concentrations of metformin on osteoclast differentiation and apoptosis and its mechanism. Pharmazie. 2021;76:244–8. 34078517 10.1691/ph.2021.1378

[27] Nayak L, Goduni L, Takami Y, et al. Kruppel-like factor 2 is a transcriptional regulator of chronic and acute inflammation. Am J Pathol. 2013;182:1696–704. 23499374 10.1016/j.ajpath.2013.01.029 PMC3644709

[28] Yan Z, Deng Y, Jiao F, Guo J, Ou H. Lipopolysaccharide downregulates kruppel-like factor 2 (KLF2) via inducing DNMT1-mediated hypermethylation in endothelial cells. Inflammation. 2017;40:1589–98. 28578476 10.1007/s10753-017-0599-0

[29] Sun Q, Xia Y, Qin H, et al. MEF2 intervened LPS-induced acute lung injury by binding to KLF2 promoter and modulating macrophage phenotype. Int Immunopharmacol. 2022;108:108873. 35729843 10.1016/j.intimp.2022.108873

[30] Tian R, Li R, Liu Y, et al. Metformin ameliorates endotoxemia-induced endothelial pro-inflammatory responses via AMPK-dependent mediation of HDAC5 and KLF2. Biochim Biophys Acta Mol Basis Dis. 2019;1865:1701–12. 31002870 10.1016/j.bbadis.2019.04.009

[31] Ding Y, Zhou Y, Ling P, et al. Metformin in cardiovascular diabetology: a focused review of its impact on endothelial function. Theranostics. 2021;11:9376–96. 34646376 10.7150/thno.64706 PMC8490502

[32] Hasanvand A. The role of AMPK-dependent pathways in cellular and molecular mechanisms of metformin: a new perspective for treatment and prevention of diseases. Inflammopharmacology. 2022;30:775–88. 35419709 10.1007/s10787-022-00980-6 PMC9007580

[33] Turkistani A, Al-Kuraishy HM, Al-Gareeb AI, et al. Pharmacological characterization of the antidiabetic drug metformin in atherosclerosis inhibition: a comprehensive insight. Immun Inflamm Dis. 2024;12:e1346. 39092773 10.1002/iid3.1346 PMC11295104

[34] He LH, Gao JH, Yu XH, et al. Artesunate inhibits atherosclerosis by upregulating vascular smooth muscle cells-derived LPL expression via the KLF2/NRF2/TCF7L2 pathway. Eur J Pharmacol. 2020;884:173408. 32739175 10.1016/j.ejphar.2020.173408

[35] Wu W, Geng P, Zhu J, et al. KLF2 regulates eNOS uncoupling via Nrf2/HO-1 in endothelial cells under hypoxia and reoxygenation. Chem Biol Interact. 2019;305:105–11. 30928399 10.1016/j.cbi.2019.03.010

[36] Fledderus JO, Boon RA, Volger OL, et al. KLF2 primes the antioxidant transcription factor Nrf2 for activation in endothelial cells. Arterioscler Thromb Vasc Biol. 2008;28:1339–46. 18467642 10.1161/ATVBAHA.108.165811

[37] Jha P, Das H. KLF2 in regulation of NF-kappaB-mediated immune cell function and inflammation. Int J Mol Sci. 2017;18:2383. 29125549 10.3390/ijms18112383 PMC5713352

[38] Chen F, Zhan J, Liu M, et al. FGF2 alleviates microvascular ischemia-reperfusion injury by KLF2-mediated ferroptosis inhibition and antioxidant responses. Int J Biol Sci. 2023;19:4340–59. 37705747 10.7150/ijbs.85692 PMC10496511

[39] Gong Z, Shu Z, Zhou Y, Chen Y, Zhu H. KLF2 regulates stemness of human mesenchymal stem cells by targeting FGFR3. Biotech Histochem. 2023;98:447–55. 37381732 10.1080/10520295.2023.2225225

[40] Wu JC, Sun J, Xu JC, Zhou ZY, Zhang YF. Down-regulated microRNA-199a-3p enhances osteogenic differentiation of bone marrow mesenchymal stem cells by targeting Kdm3a in ovariectomized rats. Biochem J. 2021;478:721–34. 33410908 10.1042/BCJ20200314

[41] Hou Z, Wang Z, Tao Y, et al. KLF2 regulates osteoblast differentiation by targeting of Runx2. Lab Invest. 2019;99:271–80. 30429507 10.1038/s41374-018-0149-x

[42] Long Z, Dou P, Cai W, Mao M, Wu R. MiR-181a-5p promotes osteogenesis by targeting BMP3. Aging (Albany NY). 2023;15:734–47. 36734882 10.18632/aging.204505 PMC9970307

[43] Xu X, Feng J, Lin T, Liu R, Chen Z. miR-181a/MSC-loaded nano-hydroxyapatite/collagen accelerated bone defect repair in rats by targeting ferroptosis pathway. J Funct Biomater. 2024;15:385. 39728185 10.3390/jfb15120385 PMC11677460

[44] Liu J, Chang X, Dong D. MicroRNA-181a-5p curbs osteogenic differentiation and bone formation partially through impairing Runx1-dependent inhibition of AIF-1 transcription. Endocrinol Metab (Seoul). 2023;38:156–73. 36604945 10.3803/EnM.2022.1516 PMC10008668

[45] Guo X, Zhang J, Han X, Wang G. LncRNA SNHG1 delayed fracture healing via modulating miR-181a-5p/PTEN axis. J Invest Surg. 2022;35:1304–12. 35263556 10.1080/08941939.2022.2048926

